# Carabid beetles of the environs of Lake Elton: fauna, population dynamics, demography

**DOI:** 10.3897/BDJ.8.e58297

**Published:** 2020-11-10

**Authors:** Kirill Makarov, Andrey Matalin

**Affiliations:** 1 Moscow State Pedagogical University, Moscow, Russia Moscow State Pedagogical University Moscow Russia; 2 Pirogov Russian National Research Medical University, Moscow, Russia Pirogov Russian National Research Medical University Moscow Russia

**Keywords:** Carabidae, Lake Elton, annual observation, semi-desert, local fauna, community structure, local population, demographic structure, wing polymorphism

## Abstract

**Background:**

The present paper includes the results of a year-round pitfall trapping survey of ground beetles in the region of Lake Elton, Volgograd Area, Russia. The main objectives of the project lie in studying the local fauna of Carabidae in the Lake Elton region, as well as their demographic structure and dispersal potential of the local populations of particular carabid species. A total of ten model habitats: six zonal (characteristic of that particular biogeographic area) and four azonal (present in a variety of biogeographical areas) were studied. In each model habitat, ten pitfall traps were set from 10 May 2006 until 10 May 2007 and were checked at 10-day intervals except for the period with negative temperatures (from 1 November 2006 until 31 March 2007). During the period of observation, 51,314 specimens of Carabidae, belonging to 149 species, were trapped. The resulting sampling-event dataset includes 24,291 plot-based observations (= sampling events), some of them containing zero records of particular species in a certain habitat and time.

**New information:**

This is the first sampling-event dataset of a year-round pitfall trapping survey (from May 2006 until May 2007) of ground-beetle communities and the demographic structure of local populations of particular species in the Lake Elton region, Volgograd Area, Russia.

## Introduction

**Figure 1.**
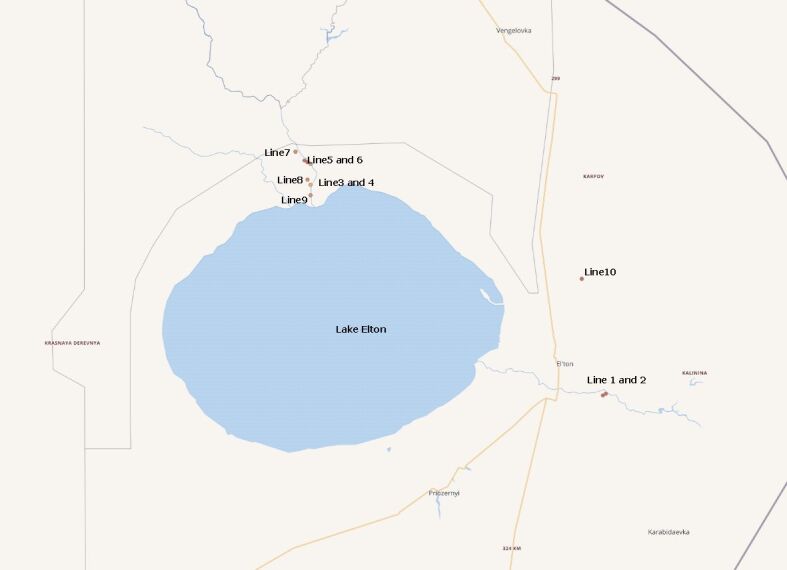
Map of the sampling sites in Lake Elton region.

The fauna of ground beetles (Carabidae) of the Lower Volga Region is well studied and includes at least 460 species (Kalyuzhnaya et al. 2000, Komarov 2002, Komarov 2020). However, information about their habitat distribution is scant, while data on the development of particular species are almost absent (Matalin and Makarov 2006, Matalin and Makarov 2008). Along a gradient of variable natural conditions within the Lower Volga Region, the landscapes surrounding the hyperhaline Lake Elton are very special. Collecting ground beetles and making information on the dynamic abundance (estimated by the number of individuals caught in soil traps per species over a certain period of time) public and available, as well as studies on the demographic structure of local populations of particular species occurrences in the most typical habitats of the Lake Elton Region (Fig. 1), were the main objectives of our work. Fieldwork was carried out in 2006-2007, with the data converted in the Darwin Core format in 2020.

## Sampling methods

### Study extent

Pitfall traps were set in ten model habitats: six zonal, characteristic of this particular biogeographic area and four azonal, present in a variety of biogeographic areas.

**Figure 2.**
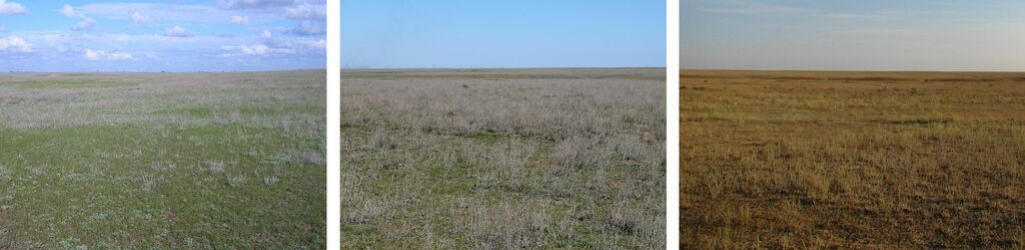
Sagebrush desert steppe (line 1) in spring, summer and in autumn.

Line 1 (49°06'44.40"N, 46°52'38.40"E) – Sagebrush desert steppe on kastanozems (= mollisols) on the left bank of Bol’shaya Smorogda River in the “Otgonnyi” natural landmark, with predominance of *Artemisialerchiana* (Fig. 2).

**Figure 3.**
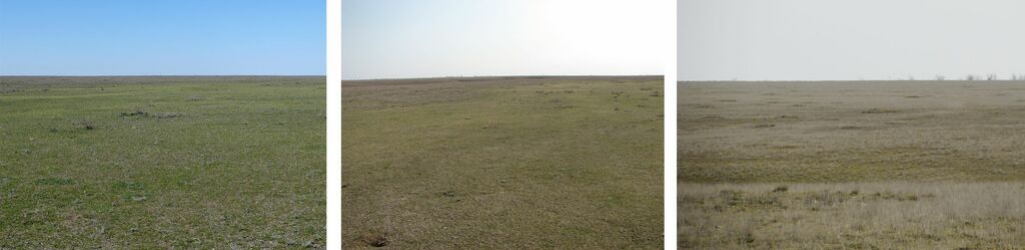
Sagebrush-grassland desert steppe (line 2) in spring, summer and in autumn.

Line 2 (49°06'42.67"N, 46°52'37.25"E) – Sagebrush-grassland desert steppe on kastanozems (= mollisols) on the left bank of Bol’shaya Smorogda River in the “Otgonnyi” natural landmark, with predominance of *Artemisialerchiana* and *Poabulbosa* (Fig. 3).

**Figure 4.**
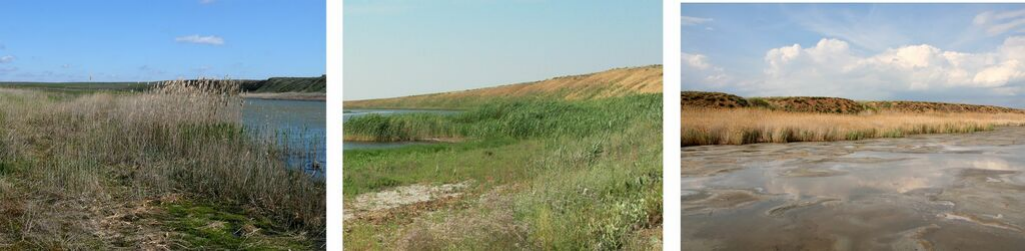
Reedbeds (line 3) in spring, summer and in autumn.

Line 3 (49°12'42.60"N, 46°39'49.80"E) – Reedbeds in a floodplain on the right bank of Khara River (3 km upstream of the mouth), with predominance of *Phragmitescommunis*, *Salsolacollina* and *S.tragus* (Fig. 4).

**Figure 5.**
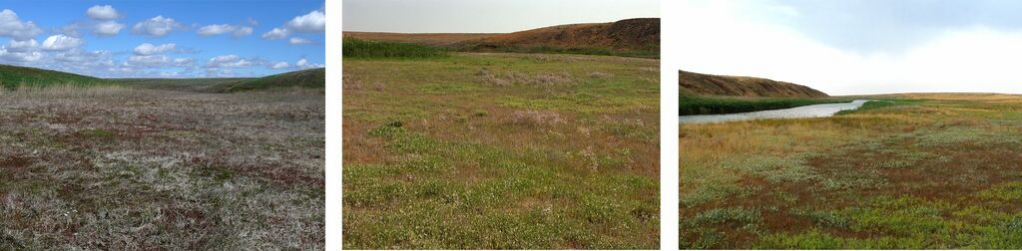
Salina on floodplain terrace (line 4) in spring, summer and in autumn.

Line 4 (49°13'19.80"N, 46°39'45.00"E) – Salina on a floodplain terrace on the right bank of Khara River (3.5 km upstream of the mouth), with predominance of *Halocnemumstrobilaceum*, *Salicorniaprostrata*, *Salsolacollina*, *S.tragus* and *Limoniumgmelinii* (Fig. 5).

**Figure 6.**
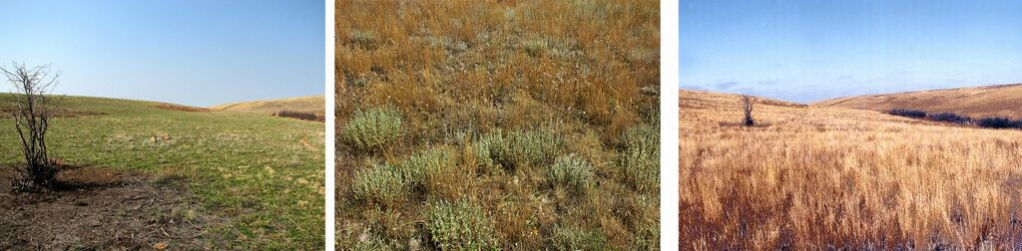
Grass-forb steppe (line 5) in spring, summer and in autumn.

Line 5 (49°13'22.28"N, 46°39'38.77"E) – Grass-forb steppe on kastanozems (= mollisols) on slopes of floodplain terraces on the right bank of Khara River (4 km upstream of mouth), with predominance of *Agropyrondesertorum* (Fig. 6).

**Figure 7.**
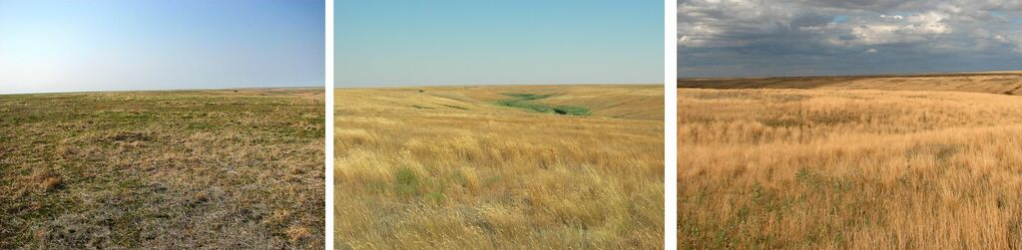
Sagebrush-grassland desert steppe (line 6) in spring, summer and in autumn.

Line 6 (49°13'24.60"N, 46°39'29.41"E) – Sagebrush-grassland desert steppe on kastanozems (= mollisols) on the watershed of Khara and Lantsug rivers (4.5 km upstream of the mouth of Khara River), with predominance of *Artemisialerchiana*, *Agropyrondesertorum*, *Anisanthatectorum* and *Anabasissalsa* Fig. 7.

**Figure 8.**
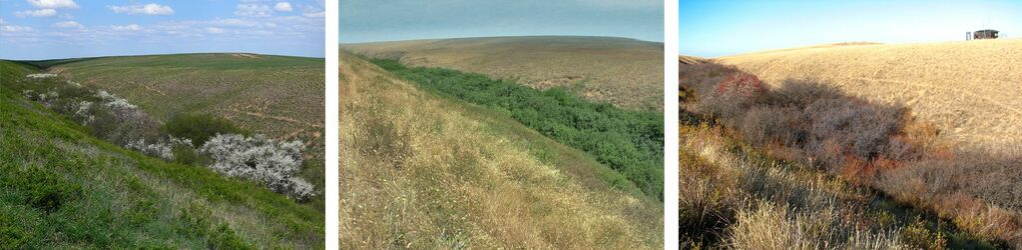
Ravine wood (line 7) in spring, summer and in autumn.

Line 7 (49°13'41.40"N, 46°39'10.80"E) – Riverine wood on kastanozems (= mollisols) on the right bank of Khara River in the “Biologicheskaya” ravine, with predominance of *Prunusspinosa*, *Rhamnuscathrtica*, *Spiraeahypericifolia*, *Rosa canina* and *Amygdalusnana* Fig. 8.

**Figure 9.**
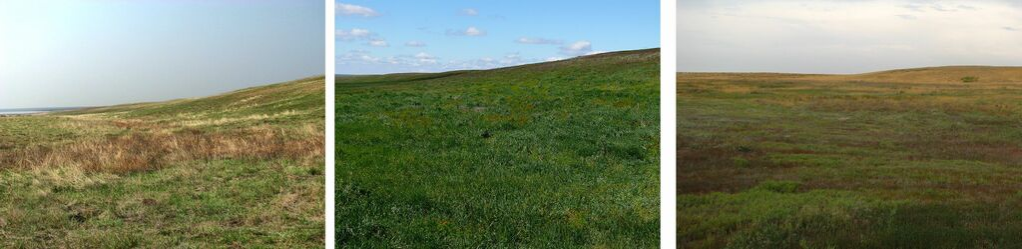
Grass-forb steppe (line 8) in spring, summer and in autumn.

Line 8 (49°12'52.20"N, 46°39'43.20"E) – Grass-forb steppe on kastanozems (= mollisols) in a depression at the bottom of a Khara River floodplain terrace (3 km upstream of the mouth), with predominance of *Stipasareptana* and *Amygdalusnana* Fig. 9.

**Figure 10.**
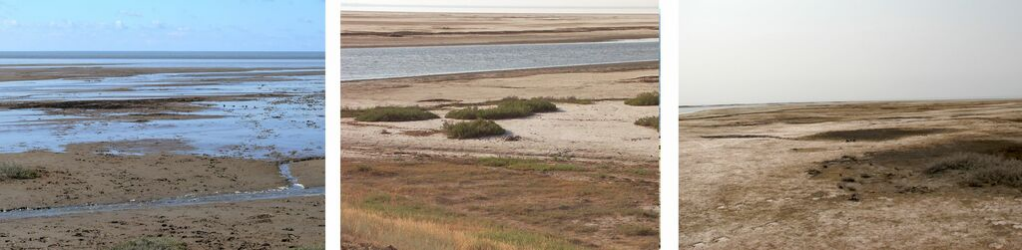
Periodically-flooded lakeside salt-marsh (line 9) in spring, summer and in autumn.

Line 9 (49°12'23.40"N, 46°39'46.80"E) – Periodically-flooded salt-marsh with mineral hydrogen-sulphide clays on the bank of Lake Elton near the mouth of the Khara River, with predominance of *Anabasissalsa* Fig. 10.

**Figure 11.**
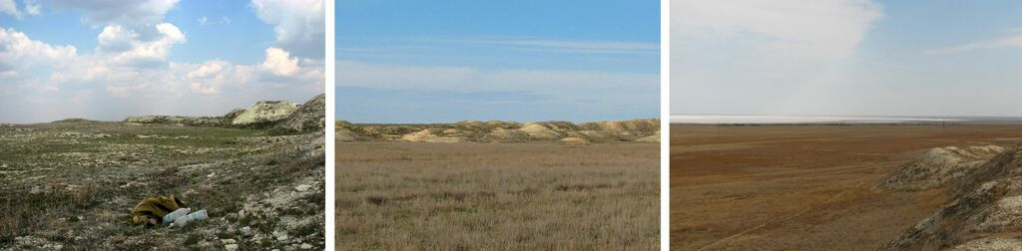
Sagebrush-grassland desert steppe (line 10) in spring, summer and in autumn.

Line 10 (49°10'3.00"N, 46°51'39.00"E) – Sagebrush-grassland desert steppe on kastanozems (= mollisols) on the northern slope of Ulagan Mountain, with predominance of *Artemisialerchiana* and *Agropyrondesertorum* (Fig. 11, Kalyuzhnaya et al. 2000).

### Sampling description

The demographic structure of the local populations of particular ground beetle species was studied in ten model habitats described above. Plastic pitfall traps of 0.5 l capacity and 95 mm upper diameter containing 4% formalin as a fixative were used. In each habitat, ten traps were arranged along a transect at 10 m intervals. Due to the high daily air temperature and low air humidity, the fixative content was increased to 3/4 of the trap’s volume. The traps were set from 10 May 2006 until 10 May 2007 and were checked at 10-day intervals on the 10^th^, 20^th^ and 30^th^(31^st^) day of each month with the exception of the period with negative temperatures, from 1 November 2006 until 31 March 2007 (Makarov and Matalin 2009).

Based on gonad condition, as well as on the degree of wear-and-tear of the mandibles, claws and cuticle, six physiological states were distinguished in the adults of both sexes: teneral, immature, mature of the parental and ancestral generations and spent of the parental and ancestral generations (Matalin and Makarov 2011).

**Figure 12.**
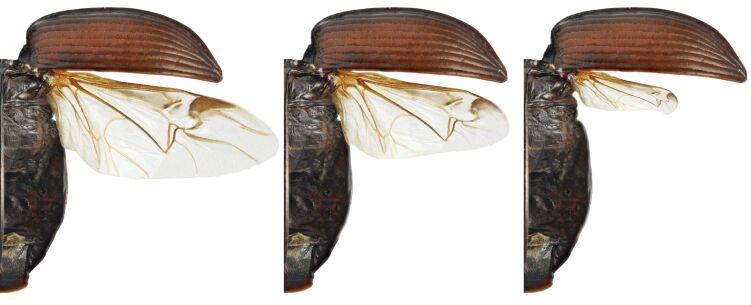
Development of hind wings in carabid beetles (macropterous, brachypterous, apterous).

The life cycle of particular species was reconstructed according to the chorological series in each local population. In ‘spring breeders’, such chronological series are represented by: immature of parental generation after hibernation → mature of parental generation → spent of the parental generation → teneral of a new generation → immature of the new generation prior to hibernation (Figure 1 in Matalin and Makarov 2011). In ‘autumn breeders’, the chronological series is as follows: teneral of the parental generation → immature of the parental generation prior to aestivation → immature of the parental generation after aestivation → mature of the parental generation → spent of the parental generation prior to hibernation (Figure 1 in Matalin and Makarov 2011). In other ‘autumn breeders’, the same order of physiological conditions of the adults was observed, but without an aestivation parapause. The dispersal capacities of individual species were estimated according to the condition of hind wings and wing muscles. We recognised macropterous, brachypterous and apterous species (Fig. 12), as well as dimorphic species when in the local population both macro- and brachy-/apterous specimens were found. Moreover, in all species, three states of wing muscles were observed: functional (100% muscle-fibres are developed), non-functional (less than 75% muscle-fibres are developed) and absent (less than 15% muscle-fibres are developed).

## Geographic coverage

### Description

Lake Elton is situated inside the blind drainage Botkul-Bulukhta Desert Depression, which belongs to the Caspian Lowland (Fig. 1). The banks of Lake Elton are located 4.2-9.5 m below sea-level. A strongly-pronounced salt-dome structure is characteristic of this region. The largest salt-domes are located on the eastern (Ulagan Mountain, altitude 68.0 m) and western (Presnyi Liman Hills, altitude 43.6 m) lakesides. Seven rivers discharge into Lake Elton: Khara, Solyanka, Chernavka and Lantsug from the northwest and Karantinka, Bol’shaya and Malaya Smorogda from the southeast (Nekrutkina 2006). All these rivers are characterised by high levels of water mineralisation which range from 0.3 to 35.6 g/l (Gorelov et al. 2006). The study area is located at the borders between several natural-climatic zones. Thus, its landscape-zonal typology is still debated. According to some authors (Safronova 2006), this general area belongs to the steppe zone but, according to others (Sapanov and Gabdullin 2006), to the semi-desert belt. Despite this, desert steppes are typical plant associations in most of the habitats; on salinas in floodplain terraces and in lakeside salt-marshes, hyper-halophilic communities are formed; dense reed-beds occur in the river valleys; in gullies on lakesides, there are trees and shrubs (Safronova 2006). Near the village of Elton, all desert steppes have been destroyed or transformed into pastures and some of these have been developed into fallow lands of different ages.

### Coordinates

49.111853 and 49.228167 Latitude; 46.653000 and 46.877333 Longitude.

## Taxonomic coverage

### Description

Ground beetles (Carabidae, include Cicindelinae) were studied during the survey. The taxonomic coverage includes representatives of 22 tribes: Harpalini – 12 genera, Cicindelini and Sphodrini – four genera each, Pogonini and Platynini – three genera each, Carabini, Pterostichini, Zuphiini and Brachinini – two genera each, Bembidini, Broscini, Callistini, Clivinini, Dyschiriini, Elaphrini, Lebiini, Notiophilini, Oodini, Scaritini, Tachyini, Trechini, Zabrini – one genus each. In general, we recorded one-third of the ground beetle fauna of the Lower Volga Region (Kalyuzhnaya et al. 2000).

## Temporal coverage

### Notes

We sampled carabids from 10 May 2006 until 10 May 2007, checking the traps at 10-day intervals, with the exception of the period with negative temperatures.

## Usage licence

### Usage licence

Creative Commons Public Domain Waiver (CC-Zero)

## Data resources

### Data package title

Carabid beetles in the environs of Lake Elton: fauna, population dynamics, demography

### Resource link

http://gbif.ru:8080/ipt/resource?r=elton-carabids

### Alternative identifiers

https://www.gbif.org/dataset/81a55a32-e89b-4959-8e49-0481c4d31973

### Number of data sets

1

### Data set 1.

#### Data set name

Carabid beetles in the environs of Lake Elton: fauna, population dynamics, demography

#### Data format

Darwin Core

#### Character set

UTF-8

#### Download URL

https://www.gbif.org/dataset/81a55a32-e89b-4959-8e49-0481c4d31973

#### Data format version

1.0

#### Description

The dataset includes two related tables of Darwin Core format, the basic *Event* table and the related *Occurrence* table (Makarov and Matalin 2020).

The *Event* table includes 10 fields and 210 records. The fields include descriptions of habitats, geography, date and subplot size. All observations were registered including those with absent records.

The table *Occurrence* includes 18 fields and 24,291 records. The fields include the scientific name and the number of males and females within each trapping event on a particular date. The two tables are related by the eventID. Version 1.0 of the table *Occurrence* contains only the number of specimens of each sex. Information on the reproductive condition, as well as the hind wing size and the wing muscles conditions of particular species will be completed in the next version of this table (see columns “reproductiveCondition”, “wingSize” and “wingMuscle”).

**Data set 1.** 

Column label	Column description
eventID	Unique identifier of each pitfall trap examination in each trap line (*Event* and *Occurrence* tables)
eventDate	Date of pitfall trap examination (*Event* and *Occurrence* tables)
samplingProtocol	pitfall traps in all records (*Event* table)
samplingEffort	100 or 110 traps per day (*Event* table)
sampleSizeValue	number of traps, 10 in all cases (*Event* table)
sampleSizeUnit	traps (*Event* table)
decimalLatitude	Geographic latitude (*Event* table)
decimalLongitude	Geographic longitude (*Event* table)
geodeticDatum	Geodetic datum, WGS84 in all records (*Event* table)
countryCode	Country code, RU in all records (*Event* table)
occurrenceID	Unique identifier of a particular observations of each species within a trapping (*Occurrence* table)
basisOfRecord	Basis of record (human observation in all records) (*Occurrence* table)
scientificName	Scientific name, including author and year (*Occurrence* table)
taxonRank	species or subspecies (*Occurrence* table)
kingdom	Animalia (in all records, *Occurrence* table)
phylum	Arthropoda (in all records, *Occurrence* table)
class	Insecta (in all records, *Occurrence* table)
order	Coleoptera (in all records, *Occurrence* table)
family	Carabidae (in all records, *Occurrence* table)
genus	Generic name (*Occurrence* table)
individualCount	number of specimen (*Occurrence* table)
organismQuantity	ind/100 trap-days (*Occurrence* table)
sex	male or female (*Occurrence* table)
reproductiveCondition	The reproductive condition of the biological individual(s) as presented in Occurrence (teneral, immature, mature, spent); planned in version 1.1
wingSize	size of hind wing, three states (normal, brachypterous, reduced); planned in version 1.1
wingMuscle	state of flying musculature (fully developed, partially developed, poorly developed); planned in version 1.1
lifeStage	imago in all cases (*Occurrence* table)
organismQuantityType	ind/100 trap-days in all records (*Occurrence* table)
occurrenceStatus	absent or present (*Occurrence* table)
specificEpithet (*Occurrence* table)	The name of the first or species epithet of the scientificName
infraspecificEpithet (*Occurrence* table)	The name of the lowest or terminal infraspecific epithet of the scientificName
coordinateUncertaintyInMetres (*Event* table)	The horizontal distance (in metres) from the given decimalLatitude and decimalLongitude describing the smallest circle containing the whole of the Location.
